# Modified Glomerular Filtration Rate-Estimating Equations Developed in Asiatic Population for Chinese Patients with Type 2 Diabetes

**DOI:** 10.1155/2014/521071

**Published:** 2014-03-05

**Authors:** Xun Liu, Xilian Qiu, Chenggang Shi, Hui Huang, Jianhua Huang, Ming Li, Tanqi Lou

**Affiliations:** ^1^Division of Nephrology, Department of Internal Medicine, The Third Affiliated Hospital of Sun Yat-sen University, Guangzhou 510630, China; ^2^College of Biology Engineering, South China University of Technology, Guangzhou, China; ^3^Department of Laboratory Medicine, The First Affiliated Hospital of Sun Yat-sen University, Guangzhou, China; ^4^Department of Cardiology, Sun Yat-sen Memorial Hospital of Sun Yat-sen University, Guangzhou, China; ^5^Department of Laboratory Medicine, The Third Affiliated Hospital of Sun Yat-sen University, Guangzhou 510630, China

## Abstract

*Objectives*. To evaluate eight modified equations developed in Asiatic populations in type 2 diabetic patients in China. *Methods*. A total of 209 Chinese patients with type 2 diabetes were recruited. Using the technetium—99m diethylenetriaminepentaacetic acid—glomerular filtration rate (GFR) to act as the reference, comparisons of their efficiency to estimate GFR in the subjects were made between various equations. *Results*. Median of difference of the Chinese equation 1 was the lowest (median of difference, 0.51 mL/min/1.73 m^2^). Median percent of absolute difference of the Chinese equation 2 was less than those of the other equations (26.97 versus ranged from 32.54 to 37.61 mL/min/1.73 m^2^, [*P* < 0.001 for all]). Precision of the simplified reexpressed MDRD equation was the best (92.9 mL/min/1.73 m^2^). Accuracies of the Chinese equation 2 were greater (*P* < 0.05 for all). There was also an improvement in chronic kidney disease (CKD) stage misclassification of the Chinese equation 2 (55.0 versus ranged from 61.2 to 64.6%, [*P* < 0.001 for all]). However, the 30% accuracies of all the equations were less than 70%. *Conclusions*. Our study highlighted a limitation in the use of the above equations in the majority of Chinese diabetic subjects. A better equation is needed in order to give an accurate estimation of GFR in type 2 diabetic patients in China.

## 1. Introduction

Human health is confronted with increasing threat from diabetes, with the statistical data from the International Diabetes Federation displaying that there are about 285 million diabetic patients all over the world by now [1]. According to the 20th World Diabetes Congress, the population of diabetic patients in Asia will increase by 60% from 2007 to 2025 [2]. In Japan, a report in 2007 by the Ministry of Health, Labor, and Welfare showed that the estimation of the number of diabetic patients was 22 million, or a fifth of adults [3]. Based on a national survey done in 2008, the prevalence of diabetes in China was 9.7% of the adults over the age of 20, counting for 92.4 million adults with diabetes [4].

Diabetes is associated with several complications, including nephropathy [5]. About 25–40% of diabetic patients will develop diabetic nephropathy, which is the main cause of end-stage renal disease in developed countries [6]. The estimation of kidney function is very important in diabetic subjects. Glomerular filtration rate (GFR) is the best measure of overall kidney function in health and disease [5]. GFR can be directly measured by infusion of external substances such as inulin, 51Cr-EDTA, ^99m^Tc-diethylenetriaminepentaacetic acid (DTPA), and iohexol [7]. However, such methods are troublesome and expensive, which limits their wide application. Therefore, a more convenient method is necessary. The National Kidney Foundation and the American Diabetes Association recommend that the modification of diet in renal disease (MDRD) equations can be used to assess GFR in adults [8, 9]. The MDRD Study equation is based on 6 variables: age, gender, ethnicity, and serum levels of creatinine, urea, and albumin [10]. Afterward, the original one was simplified to a 4-variable equation consisting of age, gender, ethnicity, and serum creatinine (SC) levels to enable its convenient clinical use [11, 12]. In 2006, the MDRD researchers used standardized serum creatinine (SC) values and developed the reexpressed MDRD equations [13]. Recently, the studies were extended to 8254 cases and the new chronic kidney disease epidemiology collaboration (CKD-EPI) equation was revised [14]. Ethnicity plays an important role in the estimation of GFR. Some researches consider that a coefficient should be used when the MDRD equations are applied to black individuals [10, 13, 15]. Taking this into account, Asiatic population should also have its own coefficient. To date, six GFR estimating equations, including the Asian equation [16], the Korean equation [17], the Japanese equation [18], the Thai equation [19], the Chinese equation 1 [20], and the Chinese equation 2 [21] were developed based on Asiatic population by amendment of the original MDRD equation. These modified equations seem more accurate in Asiatic population, but it has not been validated in diabetic patients, up until now. As diabetes is highly prevalent and costly, it is important to validate various modified equations in type 2 diabetic patients in China.

## 2. Materials and Methods

### 2.1. Sample Size

A power calculation suggested a minimum sample size of 198 using the method in Jones et al. [22]. The parameters used in the sample size formula were based on findings in a pilot study which enrolled a subgroup of patients from January 2006 to June 2008 in the same hospital (see Supplementary Table 1 and Supplementary Table 2 in the Supplementary Material available online at http://dx.doi.org/10.1155/2014/521071). The power of test was kept at 0.90 and level of significance at 0.01.

### 2.2. Subjects

A total of 209 Chinese patients with type 2 diabetes (120 males and 89 females) aged 61.6 ± 12.0 (30–89) years were enrolled consecutively from January 2005 through December 2009 in the third affiliated hospital of Sun Yat-sen University, China. Mean DTPA-GFR was 47.9 ± 26.1 (5.9–116.6) mL/min/1.73 m^2^. Patient characteristics were depicted in Table 1. Chronic kidney disease (CKD) was diagnosed and staged based on the kidney disease: Improving Global Outcomes (KDIGO) clinical practice guidelines [23]. For convenience, stages 1 and 2 and stages 3a and 3b, as well as stages 4 and 5, were combined, respectively. Exclusion criteria include patients with acute kidney function deterioration, clinical edema, skeletal muscle atrophy, pleural effusion or ascites, malnutrition, amputation, heart failure, and ketoacidosis. Patients who were taking cimetidine or trimethoprim were excluded too. No subject was treated by dialysis at the time of the study. The institutional review board at the third affiliated hospital of Sun Yat-sen University approved the study. Written informed consent had been obtained before the study.

### 2.3. Measurements of Standard GFR (sGFR)

We used GFR measured by the ^99m^Tc-DTPA renal dynamic imaging method, standardized by body surface area, as the sGFR [24, 25]. ^99m^Tc-DTPA renal dynamic imaging (modified Gate's method) was measured by Millennium TMMPR SPECT using the General Electric Medical System. High correlation was shown in the comparison of renal dynamic imaging to inulin clearance, the reference standard for measuring GFR [26]. Renal imaging also showed good agreement with plasma clearance of 51Cr-EDTA [27]. The method of ^99m^Tc-DTPA renal dynamic imaging was the same as previously described [28, 29].

### 2.4. Other Measurements

SC was determined by the enzymatic method on the Hitachi 7180 autoanalyzer (Hitachi, Tokyo, Japan; reagents from Roche Diagnostics, Mannheim, Germany) according to the manufacturer' specifications and was traceable to standard reference material (SRM 967). The following data were recorded: gender, age, height, and weight at the same time.

### 2.5. Estimations of GFR

The following equations were used:

1. Asian equation [16]: GFR = 1.086 × 175 × SC^−1.154^ × Age^−0.203^ × (0.742 if patient is female)
2. Korean equation [17]: GFR = 87.832 × SC^−0.882^ × Age^0.01^ × (0.653 if patient is female)
3. Japanese equation [18]: GFR = 194 × SC^−1.094^ × Age^−0.287^ × (0.739 if patient is female)
4. Thai equation [19]: GFR = 1.129 × 175 × SC^−1.154^ × Age^−0.203^ × (0.742 if patient is female)
5. Chinese equation 1 [20]: GFR = 175 × SC^−1.234^ × Age^−0.179^ × (0.79 if patient is female)
6. Chinese equation 2 [21]: GFR = 234.96 × SC^−0.926^ × Age^−0.280^ × (0.828 if patient is female)
7. Simplified re-expressed MDRD equation [13]: GFR = 175 × SC^−1.154^ × Age^−0.203^ × (0.742 if patient is female) × (1.212 if patient is black)
8. CKD-EPI equation [14]: GFR = 141 × (SC/*κ*)^*α*^ × (0.993)^Age^ × (1.018 if patient is female) × (1.159 if patient is black)
  - *κ* = 0.7 (female) or 0.9 (male);
  - *α* = −0.329 (female and SC ≤ 0.7 mg/dL), *α* = −1.209 (female and SC > 0.7 mg/dL);
  - *α* = −0.411 (male and SC ≤ 0.9 mg/dL), *α* = −1.209 (male and SC > 0.9 mg/dL).

### 2.6. Statistical Analysis

Difference between estimated GFR (eGFR) and sGFR was defined as eGFR minus sGFR. The percent of absolute difference between eGFR and sGFR was defined as the percent of absolute value of the difference. Accuracy was measured as the percentage of eGFR not deviating more than 15%, 30%, and 50% from the sGFR. eGFR was compared with sGFR using Bland-Altman analysis. Precision was identified as the width between the 95% limits of agreement. Wilcoxon Mann-Whitndy test and *χ* ^2^ test were used to compare the difference and accuracy. Prior to this study, a pilot study was conducted in a subgroup of patients selected from January 2006 to June 2008 and showed that the Chinese equation 2 performed better than the other equations (Supplementary Table 1 and Supplementary Table 2). Therefore, we chose eGFR measured by the Chinese equation 2 as the reference against which all comparisons between equations were made. All statistical analyses were performed using SPSS software (version 11.0 SPSS, Chicago, IL, USA) and MedCalc for Windows (version 9.3.9.0 MedCalc Software, Mariekerke, Belgium).

## 3. Results

Table 2 shows that bias of the Chinese equation 1 was the lowest (median of difference, 0.51 mL/min/1.73 m^2^). Median percent of absolute difference of the Chinese equation 2 was less than those of the other equations (26.97 mL/min/1.73 m^2^ versus ranged from 31.54 to 37.61 mL/min/1.73 m^2^, *P* < 0.001 for all). 30% to 50% accuracies of the Chinese equation 2 were greater than those of the other equations (30% accuracy, 58.4% versus ranged from 38.8 to 48.3%; 50% accuracy, 79.9% versus ranged from 60.8 to 73.2%, *P* < 0.001 for all), as was 15% accuracy (33.5% versus ranged from 17.7 to 29.7%, *P* < 0.05 for all). However, none of the equations had acceptable levels of 30% accuracy (at least 70%).

**Figure 1:**
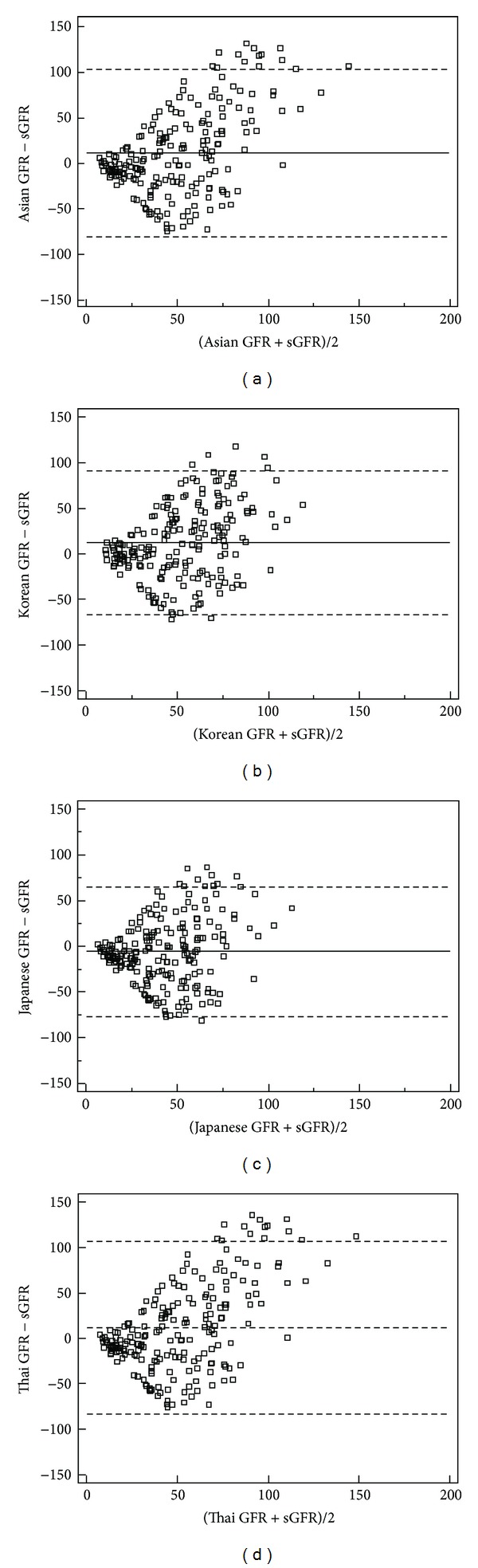
Bland-Altman plot of eGFR and sGFR (mL/min/1.73 m^2^)-1. Solid line represents the mean of difference between methods; dashed lines represent 95% limits of agreement of the mean of difference between methods. (a), (b), (c), and (d) represent the results of GFR estimated by Asian equation, Korean equation, Japanese equation, and Thai equation, respectively.

**Figure 2:**
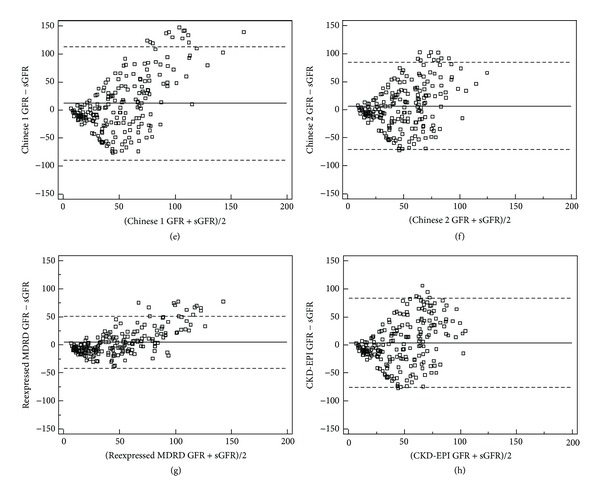
Bland-Altman plot of eGFR and sGFR (mL/min/1.73 m^2^)-2. Solid blue represents the mean of difference between methods; dashed lines represent 95% limits of agreement of the mean of difference between methods. (e), (f), (g), and (h) represent the results of GFR estimated by Chinese equation 1, Chinese equation 2, Simplified reexpressed MDRD equation, and CKD-EPI equation, respectively.

Table 3 and Figures 1 and 2 present that mean difference of the CKD-EPI equation (3.6 mL/min/1.73 m^2^) and precision of the simplified reexpressed MDRD equation (92.9 mL/min/1.73 m^2^) were the best. There was an improvement in CKD stage misclassification of the Chinese equation 2 (55.0% versus ranged from 61.2 to 64.6%, *P* < 0.001 for all). And the CKD stage misclassification of all the equations exceeded 54%.

Table 4 shows the performance of the eight equations in various stages of CKD. In CKD stages 1 and 2, median of difference of the Japanese equation was the least. Both median percent of absolute difference and accuracies of the CKD-EPI equation were better than those of the other equations. In CKD stages 3a and 3b, median of difference of the CKD-EPI equation was less than those of the other equations. In CKD stages 4 and 5, the Korean equation displayed less median of difference. The Chinese equation 2 yielded improved median percent of absolute difference and accuracies in CKD stages 3a-3b and CKD stages 4-5, as well as the CKD stage misclassification in all CKD subgroups. The performances of all the equations were progressively deteriorating with declining CKD stage.

## 4. Discussion

Diabetes is the primary cause of CKD in the USA [30]. A research by Rigalleau et al. showed that the MDRD equation was more accurate for the diagnosis and stratification of renal failure in diabetic patients [31]. The abbreviated MDRD equation [10] has been the most widely used in clinical practice, becoming a powerful screening tool for early detection of CKD. However, consensus on the most appropriate equation for Chinese diabetic patients has not got, and researches in this respect are very limited. Therefore, it is essential to undertake a study on this issue. In our study, we made comparisons between all the six modified equations developed in Asiatic population as well as the simplified reexpressed MDRD equation and the CKD-EPI equation, aiming to find out a better predictor of GFR for Chinese type 2 diabetic patients. In both the overall result and the results in different stages of CKD, GFR estimated by the Chinese equation 2 achieved the best performance. However, none of the equations had acceptable levels of 30% accuracy (at least 70%), which implied that a more accurate equation was needed to give a better prediction for Chinese type 2 diabetic patients.

So why did these equations fail to apply in type 2 diabetic patients in China and where did the bias come from?

The population studied was different. In our study, the subjects were type 2 diabetic patients in China. However, all the modified equations [16–21] as well as the simplified reexpressed MDRD equation [13] and the CKD-EPI equation [14] used to estimate GFR were established in CKD patients instead of diabetic patients, which imposed restrictions on the application of the equations. And patients studied by the Asian equation, the Korean equation, the Japanese equation, the Thai equation, the Chinese equation 1, the Chinese equation 2, and the simplified reexpressed MDRD equation were all a small part of the large population [32]. The pooled data sets in the CKD-EPI equation across various study populations and clinical conditions, which allows more general applicability than does the other equations [14]. And some differences in the performance of GFR predicting equations between various CKD as well as age subgroups were found in this study. Besides the above problems debated, ethnicity is another factor for the bias [19], which can influence the applicant of estimated equations.

The methods used to measure sGFR were different. Both in Korea and Japan, renal inulin clearance was used as the sGFR [17, 18], which was different to the method (DTPA renal dynamic imaging) used in the Chinese equation 2 [21] as well as our study and the plasma clearance of DTPA used in the Asian equation [16], the Thai equation [15]. and the Chinese equation 1 [20]. Urinary clearance of ^125^I-iothalamate was used as the sGFR in the reexpressed MDRD equation [13] and the CKD-EPI equation [14]. According to a research in 2011, underestimation of sGFR by plasma clearance of DTPA while overestimation by DTPA renal dynamic imaging were found in comparison with the inulin clearance method [26]. These could bring about bias in the estimations of sGFR in diabetic patients in China.

The calibrations of SC were different. SC levels in the Asian equation [16], the Korean equation [17], the Thai equation [19], the reexpressed MDRD equation [13], the CKD-EPI equation [14], and our study were all calibrated to an assay traceable to isotope-dilution mass spectrometry. Creatinine value was obtained by the enzyme method for the Japanese equation [18], which was calibrated to the noncompensated Jaffé method in the Cleveland Clinic laboratory in 1990. In the Chinese equation 1 [20], the SC value, which was measured by the Jaffe's kinetic method, was calibrated to the SC value measured by the Cleveland Clinic Laboratory by using a CX3 analyzer. In the Chinese equation 2 [17], the SC value was also measured by the Jaffe's kinetic method. Different ways to calibrate the data could lead to inaccuracy in equation. Variability among laboratories in the calibration of SC measurement was of critical importance in GFR estimation.

A new equation was needed to give an exact prediction of GFR in type 2 diabetic patients in China. We may take the issue discussed below into considerations.

This study displayed that the ethnicity coefficients developed in these studies might not be adequate for the management of Chinese diabetic patients, due to the difference in the calibration of SC and GFR measurement protocol and the inclusion criteria of patients. For better comparisons of different methods to estimate GFR, we had better standardize the methods to determine the value of SC and sGFR and the same inclusion criterion.

Characteristics of diabetes should be considered. None of the GFR estimated equations were based on the human physiological mechanism. They were gotten through the statistical analysis software by analyzing the data from demography. Diabetic patients were different from the ordinary CKD patients, other parameters such as the course of disease, blood-glucose level, and albuminuria, which could affect the progression of renal impairment, might be also included in the estimations of GFR. Related investigations found that many diabetic patients had a supernormal GFR before the onset of overt clinical diabetic nephropathy and progressive renal insufficiency [33–35], and the subsequent course in these patients implied that such homodynamic abnormalities may herald the development of diabetic nephropathy [36]. But study showed that MDRD was underestimated when the GFR was above or near the normal GFR [37]. We failed to find these patients, whose renal function could be restored if intervention measures were taken timely, until now. So the early detection of the supernormal GFR should not be neglected by the predicted equations.

## 5. Limitations

We had incomplete data on glycaemic status that might alter the estimation of GFR [38].

## 6. Conclusions

Our findings highlighted a limitation in the use of all the six modified equations developed in Asiatic population, as well as the simplified reexpressed MDRD equation and the CKD-EPI equation in diabetic subjects. A better equation is needed in order to give an accurate estimation of GFR for Chinese type 2 diabetic patients.

## Supplementary Material

Prior to this study, a pilot study was conducted in a subgroup of patients selected from January 2006 to June 2008. Supplementary Table 1 presented the clinical characteristics of patients. Supplementary Table 2 showed that the Chinese equation 2 performed better than the other equations.

## Figures and Tables

**Table 1:** Patient characteristics.

Sample size	209
Age (year)	61.6 ± 12.0 (30–89)
Male/female (%)	57.4/42.6
Weight (kg)	62.9 ± 11.4 (41–95)
Height (cm)	162.2 ± 8.4 (142–184)
Body surface area (m^2^)	1.66 ± 0.17 (1.28–2.15)
Body mass index (kg/m^2^)	23.9 ± 3.6 (16.4–38.2)
Serum creatinine (mg/dL)	2.6 ± 2.3 (0.4–10.9)
DTPA-GFR (mL/min/1.73 m^2^)	47.9 ± 26.1 (5.9–116.6)

CKD stages distribution	
Stage 1	14 (6.7)
Stage 2	48 (23.0)
Stage 3a	39 (18.7)
Stage 3b	46 (22.0)
Stage 4	47 (22.5)
Stage 5	15 (7.2)

DTPA-GFR: technetium—99 m diethylenetriaminepentaacetic acid—glomerular filtration rate; CKD: chronic kidney disease.

Results are expressed as mean ± SD (range) or *n* (%).

**Table 2:** Bias and accuracy between eGFR and sGFR.

	Median of difference (25% and 75% percentile)	Median % absolute difference (25%, 75% percentile)	Accuracy within		
			15%	30%	50%
Asian equation	1.12 (−9.36, 16.98)	33.83 (16.64, 60.59)*	21.5*	45.5*	66.0*
Korean equation	6.83 (−4.15, 22.75)*	34.38 (12.55, 52.74)*	29.7*	45.5*	72.7*
Japanese equation	−10.04 (−17.31, −0.47)*	33.71 (14.98, 52.70)*	24.9^‡^	45.0*	73.2*
Thai equation	2.11 (−8.68, 20.24)*	37.33 (18.66, 61.17)*	18.2*	44.0*	64.1*
Chinese equation 1	0.51 (−10.16, 22.11)^‡^	37.61 (19.06, 64.40)*	17.7*	38.8*	60.8*
Chinese equation 2	−1.28 (−6.88, 13.30)	26.97 (12.17, 44.80)	33.5	58.4	79.9
Simplified reexpressed MDRD equation	−2.83 (−11.34, 11.91)*	32.54 (15.72, 57.68)*	23.9*	44.5*	67.9*
CKD-EPI equation	−2.24 (−10.85, 11.82)*	32.79 (13.88, 54.49)*	26.8*	48.3*	69.4*

eGFR: estimated glomerular filtration rate; sGFR: standard glomerular filtration rate.

*P < 0.001 compared with Chinese equation 2-GFR.

^†^ *P* < 0.01 compared with Chinese equation 2-GFR.

^‡^ *P* < 0.05 compared with Chinese equation 2-GFR.

**Table 3:** Agreement and CKD stage misclassification between eGFR and sGFR.

	Mean of difference (bias)	Precision (levels of agreement)	CKD stage misclassification
Asian equation	9.4	184.2	61.2^†^
Korean equation	12.3	157.4	62.7*
Japanese equation	−5.9	142.0	64.6^†^
Thai equation	11.6	190.0	64.6*
Chinese equation 1	12.1	202.4	64.6*
Chinese equation 2	6.7	155.9	55.0
Simplified reexpressed MDRD equation	4.9	92.9	61.7*
CKD-EPI equation	3.6	158.6	61.7*

eGFR: estimated glomerular filtration rate; sGFR: standard glomerular filtration rate; CKD: chronic kidney disease.

**Table 4:** Performance between eGFR and sGFR in different subgroups of CKD.

	Median of difference (25% and 75% percentile)	Median % absolute difference (25% and 75% percentile)	Accuracy within			CKD stage misclassification
			15%	30%	50%	
Performance in CKD stages 1-2 (*n* = 62)						
Asian equation	20.63 (−1.57, 48.63)^∗@^	35.18 (14.47, 60.13)*	27.4*	43.5*	64.5*	56.5^∗#^
Korean equation	18.91 (−1.67, 30.96)^†&^	28.58 (10.67, 43.50)	37.1^†^	51.6*	87.1^∗#^	53.2^∗&^
Japanese equation	−7.25 (−22.04, 9.93)*	20.21 (11.53, 35.64)^#^	32.3	66.1^#^	87.1^‡^	54.8^‡#^
Thai equation	12.45 (1.06, 53.80)^∗@^	39.16 (16.89, 64.49)*	21.0*	41.9*	59.7*	61.3^∗#^
Chinese equation 1	27.43 (2.89, 63.99)^∗@^	42.13 (18.49, 70.53)*	17.7^†^	38.7*	56.5*	61.3^∗#^
Chinese equation 2	9.72 (−4.71, 29.98)^#^	23.48 (9.42, 38.95)	40.3	59.7	85.5	53.2
Simplified reexpressed MDRD equation	13.01 (−6.85, 38.11)^∗&^	27.36 (11.74, 50.44)*	30.6*	53.2*	75.8*	56.5*
CKD-EPI equation	10.06 (−4.94, 26.66)^&^	19.52 (8.12, 44.31)^#^	40.3*	64.5*	88.7^∗&^	54.8^∗#^
Performance in CKD stages 3a-3b (*n* = 85)						
Asian equation	1.72 (−9.92, 14.04)	27.78 (15.44, 55.86)*	23.5^‡^	52.9^‡^	71.8^†^	74.1^‡^
Korean equation	10.72 (−3.64, 23.35)*	36.81 (13.90, 53.44)^†^	25.9	42.4*	69.4	75.3
Japanese equation	−12.02 (−21.52, −2.49)*	32.58 (13.57, 46.99)	29.4	47.1^‡^	77.6	72.9
Thai equation	3.99 (−8.83, 16.10)^‡^	29.66 (16.28, 56.38)*	23.5^‡^	50.6^†^	69.4^†^	78.8^†^
Chinese equation 1	0.98 (−11.56, 14.97)	32.45 (17.48, 58.97)*	20.0^†^	47.1*	70.6^†^	76.5^†^
Chinese equation 2	1.28 (−8.90, 12.10)	24.28 (12.17, 43.60)	34.1	62.4	81.2	65.9
Simplified reexpressed MDRD equation	−2.42 (−12.90, 9.27)*	26.92 (14.145, 6.82)*	27.1	54.1	74.1^‡^	71.8
CKD-EPI equation	−0.54 (−12.53, 11.77)*	28.72 (14.35, 54.64)*	27.1	55.3	70.6^†^	71.8
Performance in CKD stages 4-5 (*n* = 62)						
Asian equation	−5.87 (−10.49, 1.04)^∗#^	46.04 (25.29, 63.40)^∗#^	12.9	37.1^†^	59.7*	48.4^∗&^
Korean equation	0.93 (−5.03, 10.03)^ ∗#^	34.48 (13.27, 65.66)	27.4	43.5*	62.9*	54.8^∗#^
Japanese equation	−8.22 (−12.54, −3.35)*	48.35 (33.95, 65.14)^‡@^	11.3^&^	21.0^&^	53.2^&^	62.9*
Thai equation	−5.56 (−10.26, 1.62)^∗#^	43.91 (23.56, 62.84)*	8.1^‡#^	37.1*	61.4*	51.6^∗@^
Chinese equation 1	−6.41 (−11.55, −0.47)^∗#^	49.28 (27.83, 66.21)^†^	14.5	27.4^#^	51.6^†#^	51.6^∗&^
Chinese equation 2	−1.95 (−6.94, 5.16)	29.43 (14.67, 52.26)	25.8	51.6	72.6	41.9^&^
Simplified reexpressed MDRD equation	−6.48 (−11.30, −0.21)*	46.90 (30.80, 64.90)^‡&^	12.9^&^	22.6^@^	51.6^†&^	53.2^∗#^
CKD-EPI equation	−6.90 (−11.56, −0.47)*	50.08 (32.79, 66.25)^†&^	12.9^#^	22.6^@^	48.4^†&^	54.8^∗#^

eGFR: estimated glomerular filtration rate; sGFR: standard glomerular filtration rate; CKD: chronic kidney disease.

*P < 0.001 compared with Chinese equation 2-GFR.

^†^ *P* < 0.01 compared with Chinese equation 2-GFR.

^‡^ *P* < 0.05 compared with Chinese equation 2-GFR.

^@^ *P* < 0.001 compared with the subgroup with CKD stages 3a-3b.

^&^ *P* < 0.01 compared with the subgroup with CKD stages 3a-3b.

^#^ *P* < 0.05 compared with the subgroup with CKD stages 3a-3b.
