# Supplementary material for: Modified Glomerular Filtration Rate-Estimating Equations Developed in Asiatic Population for Chinese Patients with Type 2 Diabetes
Source: Int J Endocrinol. 2014 Mar 5;2014:521071. doi: 10.1155/2014/521071 (PMC3966408; doi:10.1155/2014/521071)
Supplement: Supplementary file 1 — Prior to this study, a pilot study was conducted in a subgroup of patients selected from January 2006 to June 2008. Supplementary Table 1 presented the clinical characteristics of patients. Supplementary Table 2 showed that the Chinese equation 2 performed better than the other equations. [file 521071.f1.pdf]

Supplementary Appendix

Supplement to: Modified glomerular filtration rate-estimating equations developed in Asiatic population for Chinese patients with type 2 diabetes

Xun Liu, Xilian Qiu, Chenggang Shi, Hui Huang, Jianhua Huang, Ming Li and Tanqi Lou.

Table of Contents

List of Supplementary Tables 2

Table S1. Clinical characteristics in subgroup of patients selected from January 2006 to June 2008... 3

Table S2. Performance between eGFR and sGFR in subgroup of patients selected from January 2006 to June 2008........................... 4

Table S1. Clinical characteristics in subgroup of patients selected from January 2006 to June 2008

Sample size	67
Age(year)	60.3±12.6 (37-85)
Male/female (%)	68.7/31.3
Weight(kg)	64.7±10.7 (43-95)
Height(cm)	163.7±7.8 (145-180)
Body surface area (m^2)	1.70±0.16 (1.34-2.15)
Body mass index (kg/m^2)	24.0±2.9 (18.8-34.7)
Serum creatinine (mg/dL)	2.6±2.4 (0.4-10.8)
DTPA-GFR($ml/min/1.73m^2$)	50.3±25.9 (7.8-114.0)
CKD stages distribution	
Stage 1	6(9.0)
Stage 2	16(23.9)
Stage 3a	15(22.4)
Stage 3b	13(19.4)
Stage 4	12(17.9)
Stage 5	5(7.5)

DTPA-GFR: technetium-99m diethylenetriaminepentaacetic acid-glomerular filtration rate; CKD: chronic kidney disease

Results are expressed as mean ± SD (range) or n (%)

Table S2. Performance between eGFR and sGFR in subgroup of patients selected from January 2006 to June 2008

	Median of difference	Median % Absolute difference	Accuracy within			CKD stage misclassification
			15%	30%	50%	
Asian equation	-7.4	33.2	29.9	43.3	67.2	62.7
Korean equation	10.7	37.8	25.4	40.3	68.7	62.7
Japanese equation	-7.4	33.2	29.9	43.3	67.2	62.7
Thai equation	2.9	39.9	20.9	41.8	67.2	62.7
Chinese equation 1	0.5	37.6	22.4	37.3	64.2	64.2
Chinese equation 2	2.0	25.1	31.3	59.7	76.1	55.2
Simplified re-expressed MDRD equation	-0.4	33.1	26.9	47.8	67.2	61.2
CKD-EPI equation	-0.4	29.9	29.9	50.7	68.7	58.2

eGFR: estimated glomerular filtration rate; sGFR: standard glomerular filtration rate; CKD: chronic kidney disease
